# An Analysis of the Sources of Ultrafine Particles During Severe Haze Pollution Periods in China

**DOI:** 10.3390/toxics14070588

**Published:** 2026-07-03

**Authors:** Jingkun Zhou, Long Sun, Yunkai Zhou

**Affiliations:** 1 School of Business, Ludong University, Yantai 264025, China; zhoujingkun@163.com (J.Z.);; 2 Adam Smith Business School, University of Glasgow, Glasgow G12 8QQ, UK

**Keywords:** haze pollution, ultrafine particles, pollution-intensive industries, carrying capacity for pollution-intensive industries, desulfurization and denitrification, electrostatic dust removal

## Abstract

Haze Pollution in China arises from the rapid enlargement of ultrafine particles into light-absorbing fine particulate matter through adsorption processes under atmospheric stagnation conditions. This study focuses on the sources of ultrafine particles (UFPs), the most critical component of haze pollutants during severe pollution periods in China. Utilizing methods including the spatial Durbin model and statistical data for the 28 cities (the “2 + 26” cities) within the Beijing–Tianjin–Hebei air pollution transmission channel—suffering the most severe haze pollution—it investigates the impact of pollution-intensive industries on haze pollution. This study reveals several key findings regarding China’s haze pollution. First, the principal source of ultrafine particles within China’s haze stems from the desulfurization, denitrification, and dust removal processes of pollution-intensive industries (the direct effect of these industries on haze is 0.028 * according to the SDM regression results). Crucially, the specific operational factors driving the abrupt increase in atmospheric UFPs during severe haze periods in China are identified as extensive management practices in desulfurization, the progressive tightening and annual escalation of denitrification emission standards, and the reliance on electrostatic precipitation which is ineffective against ultrafine particles. Second, haze pollution predominantly occurs in regions characterized by concentrations of pollution-intensive industries coupled with weak atmospheric environmental self-purification capacity (this carrying capacity for pollution-intensive industries exerts a significant negative impact on haze, demonstrated by a direct effect of −0.020 **; further analysis reveals that this is caused by regional differences in atmospheric self-purification capacity). Third, regional air transport acts as a contributing source, introducing UFPs from neighboring areas into local haze pollution, reflected by an indirect effect of pollution-intensive industries of 0.151 ** stemming from such spatial spillovers. Based on these conclusions, the study proposes a set of policy recommendations: relocate pollution-intensive industries using a gradient approach based on atmospheric self-purification capacity differences; systematically upgrade wet flue gas desulfurization technologies for industrial emissions; effectively promote technological innovation in denitrification processes; implement scientific controls on ammonia emissions; strengthen R&D in core technologies for UFP removal; innovate dust removal technologies to enhance overall system efficiency; reinforce regional coordinated governance; implement targeted training programs and select qualified management personnel; systematically enhance the environmental management capabilities of staff; and effectively mitigate the spillover effects of haze pollution.

## 1. Introduction

### 1.1. Background

At the National People’s Congress in March 2017, as well as at multiple State Council executive meetings and through authoritative media outlets, Premier Li Keqiang emphasized that if any research team could fully elucidate the formation mechanisms and health impacts of haze and propose effective solutions, “those who achieve breakthroughs will be generously rewarded” [1]. This statement was widely reported by authoritative national media, including the Chinese Academy of Sciences, the Beijing News, People’s Daily Online, CCTV News, the Central People’s Government website (www.gov.cn), and Guangming Daily, highlighting a national-level initiative to uncover the causes of haze pollution and develop effective solutions. According to the findings of [2], the formation mechanism of haze in China can be described as follows: Under stagnant meteorological conditions, large quantities of ultrafine particles (UFPs) rapidly grow into light-absorbing fine particles through adsorption processes. With increasing emissions of water vapor from desulfurization processes in pollution-intensive industries and artificial atmospheric spraying in haze-affected regions, ambient humidity rises accordingly. This accelerates both the rate and extent of transformation of UFPs into light-absorbing fine particles. As a result, the light-extinction capacity of these particles is significantly enhanced, leading to reduced visibility and the emergence of haze pollution. As humidity continues to increase, this transformation process becomes even more rapid and pronounced, thereby intensifying haze pollution. This formation mechanism can be tested through the following experiment: Place a humidifier in a closed room. If purified water is used in the humidifier, the air quality in the room remains good after three hours. If tap water is used, the room exhibits moderate or light haze after three hours. If reclaimed water or circulating water is used (factories discharge circulating water, in which ultrafine particles are in a saturated state), the room exhibits heavy haze after three hours; if the room is relatively large, a longer time will be required. These findings suggest that, to effectively mitigate haze pollution in China, it is crucial to identify the primary sources of UFPs in the atmosphere.

### 1.2. Literature Review

Currently, particulate matter has become the primary air pollutant in most Chinese cities [3]. Particulate matter is classified based on particle size, typically measured by aerodynamic diameter, into: total suspended particles (TSP, aerodynamic diameter ≤ 100 μm), inhalable particles (PM_10_, aerodynamic diameter ≤ 10 μm), fine particles (PM_2.5_, aerodynamic diameter ≤ 2.5 μm), and ultrafine particles. “The number concentration of UFPs generally dominates the total particle number concentration in the atmosphere” [4]. Due to their small size, large specific surface area, high number concentration, and propensity to adsorb various pollutants such as heavy metals and organic compounds, ultrafine particles pose particularly severe risks to human health [5]. Ultrafine particles, as the core component of PM_2.5_ (accounting for over 95%), are characterized by their small size, large specific surface area, and strong capacity to adsorb heavy metals and organic pollutants [6,7].

Recent toxicological studies indicate that the toxicity of UFPs depends not only on their mass concentration, but also is closely related to their source, chemical composition, and physical form. For instance, Vallabani et al. (2023) systematically analyzed the relative toxicological potency of UFPs emitted from different traffic sources and found that the genotoxicity of combustion-derived particles was closely associated with their polycyclic aromatic hydrocarbon content [8]. The size of aviation-related UFPs is mainly distributed in the range of 10–20 nm, which is markedly smaller than the >50 nm typical of traffic sources; this specificity in size and composition imparts distinct health effect characteristics that differ from those of other combustion sources [9]. This highlights the urgency of refined source apportionment and physicochemical characterization of UFPs, and also echoes the strong call by the World Health Organization to prioritize research on the biological mechanisms of UFPs. However, UFPs feature complex sources, extremely small sizes, highly variable environmental concentrations, and are difficult and costly to detect; consequently, most regions have not incorporated UFPs into routine monitoring [10].

Domestic studies have similarly confirmed this dilemma: [11] conducted continuous monitoring of UFPs during high-pollution winter–spring periods in Guangzhou and found that even within the same city, significant differences existed in chemical composition and the number-to-mass concentration ratio across different time periods. The study explicitly pointed out that relying solely on conventional chemical composition analysis and mass concentration monitoring may not effectively reveal the true health hazards of UFPs with high number concentrations. In summary, research on ultrafine particles remains relatively scarce and is still in an exploratory stage.

The sources and formation mechanisms of atmospheric UFPs are extremely complex. Conventional theory emphasizes that their primary sources are mainly soot directly emitted from combustion sources (e.g., motor vehicles, biomass burning), while secondary sources are formed from gaseous precursors through atmospheric nucleation and condensational growth [12].

In urban environments in particular, the contributions of these sources are not constant but vary dramatically with time and meteorological conditions. For example, Agudelo-Castañeda et al. (2019), using long-term particle size distribution observations and k-means clustering analysis, identified eight categories of UFP sources with distinct size distribution characteristics in urban areas [13]. Moreover, recent studies have revealed that the composition and morphology of atmospheric UFPs extend far beyond the simple concept of “combustion spherules.” [14] employed combined atomic force microscopy and transmission electron microscopy techniques to successfully extract and characterize various crystalline UFPs from the atmosphere of a northern Mexican city; their morphologies included soot and crystalline structures, with sizes as small as 10 nm. Such non-spherical, poorly soluble mineral UFPs may have atmospheric residence times different from those of spheroidal particles, and their pathways of health effects may also differ from those of combustion-derived carbonaceous particles. This indicates that, in addition to gas-to-particle conversion processes, mechanical processes such as road dust resuspension, soil weathering, and industrial friction can also produce “ultrafine mineral dust.” Meanwhile, extensive chemical analyses have confirmed that ambient UFPs commonly carry toxic components such as heavy metals, polycyclic aromatic hydrocarbons, and black carbon, with their exact composition varying by emission source [15].

The complexity of UFP sources described above exhibits particular characteristics in the context of China’s air pollution control history. Some studies have pointed out that, around 2012, following the implementation of mass-based measurement standards for haze pollution control, heavily polluted regions adopted extensive measures to reduce the mass concentration of particulate matter. These included widespread spraying of water vapor containing UFPs and the large-scale application of electrostatic precipitation technologies. Critically, while electrostatic precipitation—widely adopted in China—is effective for coarse particles, it lacks efficacy in removing fine and ultrafine particles. This led to substantial low-cost emissions of UFPs [16].

Concurrently, the mandatory removal of flue gas–gas heat exchangers in thermal power plants resulted in significantly increased emissions of water vapor and particulate matter during wet flue gas desulfurization processes. The interaction of these emissions with stagnant meteorological conditions directly catalyzed severe haze episodes in regions like Beijing–Tianjin–Hebei.

In 2013, despite stricter monitoring measures (including online systems), pollution-intensive enterprises excessively and inappropriately applied ammonia injection to meet emission standards. This caused ammonia slip, triggering a rapid surge in secondary UFP formation. Consequently, haze pollution intensified rather than abated [16].

These observations indicate that pollution control technologies and regulatory measures, designed to reduce emissions, may nevertheless lead to a significant increase in ultrafine particles under specific conditions. This phenomenon is particularly prominent in desulfurization and denitrification processes widely applied in industries such as thermal power and steel production.

The desulfurization stage, while effective at removing sulfur dioxide (SO_2_) to meet emission standards, emits saturated wet flue gas containing substantial amounts of water vapor and UFPs. Upon release into the atmospheric environment, these components rapidly mix and condense with cooler air, forming numerous ultrafine particles. These particles act as “seeds for haze formation”, accumulating rapidly under stagnant meteorological conditions.

In the denitrification stage, Selective Catalytic Reduction (SCR) technology, implemented to remove nitrogen oxides (NO_x_), requires ammonia injection as a reducing agent. When the ammonia dosage exceeds reaction requirements or is poorly controlled, ammonia slip occurs. This escaped ammonia entering the atmosphere can subsequently react with residual acidic gases in the flue gas—such as sulfur dioxide (SO_2_) and sulfur trioxide (SO_3_)—triggering a gas-to-particle conversion process. This reaction generates secondary ultrafine particles, including ammonium sulfate and ammonium nitrate, which directly intensify haze pollution.

To address these issues, this study will employ methodologies including the Spatial Durbin Model (SDM), combined with statistical data from 28 cities in the Beijing–Tianjin–Hebei Air Pollution Transmission Corridor—the region in China most severely affected by haze pollution. Our research investigates the impact of pollution-intensive industries on haze formation.

Guided by fusion innovation theory, we aim to systematically identify the dominant sources of ultrafine particles—the core component of haze pollution—through multidimensional analysis across: Industrial structures, Process technologies, Management practices and Policy frameworks. The findings are expected to provide scientifically grounded and targeted policy recommendations for mitigating haze pollution in China.

## 2. Research Design

### 2.1. Model Selection

The Spatial Durbin Model is primarily used to capture mutual influences and spatial lag effects in spatial data. Its theoretical framework and methodology have been continuously refined and are widely applied in the field of environmental science. Serving as a core analytical tool for cross-regional studies of haze pollution, it provides a scientific basis for regional collaborative governance. For example, Li Yejin et al. (2024) applied the SDM to investigate carbon peaking classification and influencing factors at the county level in the Beijing–Tianjin–Hebei region [17]. Zhang Tonggong et al. (2024) conducted an in-depth analysis of the impact of business environment optimization on environmental pollution based on the SDM [18]. Their findings indicate that optimizing the business environment has a significant inhibitory effect on environmental pollution. Moreover, this positive effect can extend to surrounding areas, effectively reducing pollution levels in neighboring regions.

Regarding spatial econometric models, this study considered two commonly used specifications: the Spatial Autoregressive Model (SAR) and the Spatial Durbin Model (SDM). Advantages of the Spatial Autoregressive Model (SAR): Model simplicity and ease of interpretation; suitability for preliminary testing of spatial dependence. Disadvantages of SAR: Failure to account for spatial spillovers from independent variables; inability to decompose direct and indirect effects. Advantages of the Spatial Durbin Model: Incorporation of spatial lag terms for both the dependent and independent variables, yielding stronger explanatory power; enables decomposition into direct effects, indirect effects, and total effects; and offers greater model flexibility/inclusivity. Disadvantages of SDM: Higher model complexity and requires more technical expertise for interpretation.

In light of the above analysis, this research introduces the Spatial Durbin Model to investigate the impact of output from pollution-intensive industries on haze pollution, based on the perspective of the atmospheric carrying capacity index.

#### 2.1.1. Spatial Autoregressive Model

The spatial autoregressive (SAR) model is specified as:

$${{lnPM}_{25}}_{it}=\rho \sum_{j=1}^{N}W_{it}{{lnPM}_{25}}_{it}+\beta_{1}{carrying}_{it}+\beta_{2}{lngti}_{it}+\beta_{3}{{lnK}_{1}}_{it}+\beta_{4}{{lnK}_{2}}_{it}\;+\beta_{5}{{lnK}_{3}}_{it}+\beta_{6}{{lnK}_{4}}_{it}+\mu_{i}+\lambda_{i}+\varepsilon_{it}$$

where ρ denotes the spatial lag (autoregressive) coefficient, and $W_{it}$ represents the element in the *i*-th row and *j*-th column of the N*N row-standardized non-negative spatial weight matrix *W*. ${{lnPM}_{25}}_{it}$ denotes the natural logarithm of PM_2.5_ concentration; ${carrying}_{it}$ denotes the carrying capacity index for pollution-intensive industries; ${lngti}_{it}$ denotes the natural logarithm of green technological innovation; ${{lnK}_{1}}_{it}$, ${{lnK}_{2}}_{it}$, ${{lnK}_{3}}_{it}$ and ${{lnK}_{4}}_{it}$ represent the natural logarithms of administrative area size, urbanization level, registered urban unemployment rate, and tax policy, respectively. The subscripts i and t denote city *i* and year *t*, respectively. $\mu_{i}$ and $\lambda_{i}$ represent spatial (individual) effects and time effects, respectively.

#### 2.1.2. Spatial Durbin Model

The spatial Durbin model is specified as:

$$\begin{array}{c} {{lnPM}_{25}}_{it}=\rho \sum_{j=1}^{N}W_{it}{{lnPM}_{25}}_{it}+\beta_{1}{carrying}_{it}+\beta_{2}{lngti}_{it}+\beta_{3}{{lnK}_{1}}_{it}+\beta_{4}{{lnK}_{2}}_{it}+\beta_{5}{{lnK}_{3}}_{it}+\beta_{6}{{lnK}_{4}}_{it}\\ \;\;\;\;\;\;+\beta_{7}\sum_{j=1}^{N}W_{it}{carrying}_{it}+\beta_{8}\sum_{j=1}^{N}W_{it}{lngti}_{it}+\beta_{9}\sum_{j=1}^{N}W_{it}{{lnK}_{1}}_{it}+\beta_{10}\sum_{j=1}^{N}W_{it}{{lnK}_{2}}_{it}\\ \;\;\;\;\;\;+\beta_{11}\sum_{j=1}^{N}W_{it}{{lnK}_{3}}_{it}+\beta_{12}\sum_{j=1}^{N}W_{it}{{lnK}_{4}}_{it}+\mu_{i}+\lambda_{i}+\varepsilon_{it} \end{array}$$

where ρ denotes the spatial autoregressive coefficient, and $W_{it}$ represents the element in the *i*-th row and *j*-th column of the N*N row-standardized non-negative spatial weight matrix *W*. ${{lnPM}_{25}}_{it}$ denotes the natural logarithm of PM_2.5_ concentration; ${carrying}_{it}$ denotes the carrying capacity index for pollution-intensive industries; ${lngti}_{it}$ denotes the natural logarithm of green technological innovation; ${{lnK}_{1}}_{it}$, ${{lnK}_{2}}_{it}$, ${{lnK}_{3}}_{it}$ and ${{lnK}_{4}}_{it}$ represent the natural logarithms of administrative area size, urbanization level, registered urban unemployment rate, and tax policy, respectively. $\mu_{i}$ and $\lambda_{i}$ represent spatial (individual) effects and time effects, respectively.

### 2.2. Variable Selection

The specific variables selected in this research are presented in Table 1.

Reasons for using haze data instead of ultrafine particle data in the relevant analysis:

First, haze pollution is a phenomenon of visual obscuration. Haze is caused by a large number of particles, while the mass of the particles has a very small effect. For example, taking spherical particles as an illustration, the mass of one PM_2.5_ particle ≈ the mass of 125 PM_0.5_ particles ≈ the mass of 15,625 PM_0.1_ particles (assuming identical density). One hundred PM_0.5_ particles are equal to 80% of the mass of one PM_2.5_ particle. If these 100 PM_0.5_ particles are arranged into four layers, with 25 particles per layer, the total light-blocking area and the average thickness of the four layers of 25 PM_0.5_ particles are far greater than those of a single PM_2.5_ particle. The light-blocking effect of 100 PM_0.5_ particles, despite weighing 20% less, is far stronger than that of one PM_2.5_ particle [16]. Moreover, existing studies have found through experiments that in some regions, ultrafine particles account for a large proportion of the mass of haze pollutants. Our project team, through the above conversion, has found that the number of ultrafine particles accounts for more than 99% of the total particle number in haze pollutants, and in some cases even more than 99.9%.

Second, the objective of this study is to identify the main sources of ultrafine particles during the severe haze pollution period in China (2012–2016). The main sources of ultrafine particles in the air may differ across periods and regions. It is not accurate to identify the main sources of ultrafine particles in the past air solely through experiments, and high-altitude sampling is also not feasible.

Third, the haze data selected for this study are derived from haze satellite imagery. Based on the haze formation mechanism—ultrafine particles continuously transform into light-absorbing fine particles in the air, and this transformation accelerates with increasing humidity; under stagnant weather conditions, light-absorbing fine particles accumulate in increasing numbers, eventually forming haze pollution—the satellite images of haze can be directly converted into fine particle data, which can then be further converted into ultrafine particle data [2]. Given the complex sources of ultrafine particles, the great difficulty and high cost of monitoring, and the current lack of ultrafine particle monitoring data, the use of haze data converted from satellite imagery as a substitute for ultrafine particle monitoring data is an effective solution we have identified.

Air Pollution Index (pollution): The Air Pollution Index (API) is widely used as a core indicator for evaluating regional air pollution levels. It is typically constructed through the aggregation of multiple pollutant concentrations, including PM_2.5_, PM_10_, SO_2_, and NO_2_. By integrating information from various pollutants, the API is capable of providing a comprehensive and accurate representation of the overall impacts of air pollution on human health and the ecological environment. Yang et al. (2023) employed the API to investigate the mechanisms through which meteorological and geographical factors influence air pollution in chemical industrial parks, thereby revealing the dynamic effects of these factors on API variations [20]. In this study, the Air Pollution Index is comprehensively calculated based on the emissions of three major pollutants: sulfur dioxide emissions, nitrogen oxide emissions, and the geographical average PM_2.5_. The formula is: Air Pollution Index = (SO_2_ emissions/GDP + NO_x_ emissions/GDP + geographical average PM_2.5_/GDP)/3.

Output Value of Air Pollution-Intensive Industries: Previous research by [21], focusing on the determinants and spatial spillover effects of haze pollution in the Beijing–Tianjin–Hebei region, has identified several key findings: In cities with a strong industrial base or those undertaking industrial transfer, a higher share of the secondary industry in the economic structure has been identified as a major contributor to deteriorating air quality. In addition, significant structural issues in energy consumption, characterized by high energy intensity and high pollution emissions, have further accelerated air quality deterioration, particularly in Hebei Province. These findings indirectly suggest a strong association between the output of air pollution-intensive industries and haze pollution. Based on data from 2009, 2011, 2013, and 2015, the air pollution intensity indices for different industries in China were calculated. The six industries with the highest pollution intensity indices are: Production and Supply of Electric Power and Heat Power; Smelting and Pressing of Ferrous Metals; Petroleum Processing and Coking; Manufacture of Raw Chemical Materials and Chemical Products; Manufacture of Non-metallic Mineral Products; and Smelting and Pressing of Non-ferrous Metals. The output value of pollution-intensive industries is taken as the sum of the output values of these six industries with the highest pollution intensity indices [22].

Sales Value of Air Pollution-Intensive Industries: This variable focuses on six representative industries that are closely associated with atmospheric pollution within the industrial economic structure. It is constructed by aggregating the sales values of these six industries. The selection of these industries is based on a comprehensive consideration of their contributions to air pollutant emissions, their shares in the regional economy, and the sustainability of their development.

Carrying Capacity Index for Pollution-Intensive Industries: This variable is employed to quantify the ability of the atmospheric environment to accommodate and buffer pollutant inputs. As a complex and dynamic system, the atmosphere possesses an inherent capacity to absorb and assimilate a certain level of pollutants. This index is constructed by comprehensively incorporating the influences of atmospheric physical properties, the chemical properties of particulate matter (especially ultrafine particles), and meteorological conditions on pollutant dispersion and assimilation. Drawing on the perspectives of [23] and the evaluation indicator systems constructed by [24], [25], [26], [27], [28], and [29], this study designs an evaluation indicator system for the carrying capacity of pollution-intensive industries based on environmental self-purification capacity from four dimensions: industrial attractiveness, industrial selectivity, industrial support capacity, and industrial development capacity (see Table 2). Principal component analysis is then used to calculate the carrying capacity for pollution-intensive industries.

Green Technological Innovation (gti): This variable refers to a system of technologies that reduces pollution, lowers resource consumption, and improves ecological performance. It is characterized by a dynamic system composed of knowledge, capabilities, and material means. In this research, the level of green technological innovation is measured by the number of green patents across cities in different years.

Administrative Area Size (K_1_): This is defined as the total land and water area under the jurisdiction of an administrative unit. A larger administrative area is generally associated with a more dispersed distribution of population and industrial activities, which may contribute to the physical dilution of air pollutants. In addition, variations in area size reflect differences in resource endowments and environmental carrying capacity, thereby influencing the dispersion potential of pollutants and the effectiveness of governance strategies.

Urbanization Level (K_2_): This is a key indicator of the urbanization process and is measured by the proportion of the urban population in the total population. It reflects the degree of population agglomeration in urban areas. During the urbanization process, significant changes occur in industrial activity, energy consumption, and transportation flows, generating complex effects on the atmospheric environment. On the one hand, urbanization may increase pollution emissions; on the other hand, it may also be accompanied by improved environmental awareness and advances in pollution control technologies.

Registered Urban Unemployment Rate (K_3_): This is an important indicator for assessing economic vitality and labor market conditions. In general, lower unemployment rates are associated with higher economic activity, which may lead to increased industrial production and energy consumption, thereby affecting the atmospheric environment. In addition, overall employment conditions may influence the level of environmental investment and the priority of policy implementation by governments and society.

Tax Policy (K_4_): Tax policy can regulate corporate behavior and economic activities through fiscal instruments. The imposition of higher environmental taxes on polluting entities can increase production costs and incentivize emission reductions. Conversely, tax incentives for environmental protection industries and green technological innovation can stimulate investment and the adoption of cleaner technologies, thereby contributing to improvements in air quality.

### 2.3. Data Sources

This research selects “the Beijing–Tianjin–Hebei region and its 26 neighboring pollution-monitoring cities” in China, which constitute the urban agglomeration most severely affected by haze pollution—namely, the Beijing–Tianjin–Hebei air pollution transmission channel cities. The sample includes Beijing and Tianjin; Shijiazhuang, Tangshan, Langfang, Baoding, Cangzhou, Hengshui, Xingtai, and Handan in Hebei Province; Taiyuan, Yangquan, Changzhi, and Jincheng in Shanxi Province; Jinan, Zibo, Jining, Dezhou, Liaocheng, Binzhou, and Heze in Shandong Province; and Zhengzhou, Kaifeng, Anyang, Hebi, Xinxiang, Jiaozuo, and Puyang in Henan Province [30]. Based on the carrying capacity index for pollution-intensive industries, this research further examines the impact of output from air pollution-intensive industries on haze pollution. Due to the lack of direct monitoring data for UFPs, haze pollution data are used as a proxy for assessing UFP-related atmospheric pollution in this research. Given that large-scale administrative shutdowns and production restrictions of approximately 100,000 polluting enterprises occurred in China during 2017–2018, which may introduce structural disturbances into the analysis, the final dataset covers a panel of prefecture-level cities across 31 provinces in China over the period 2000–2018. For the analysis of ultrafine particle sources using the spatial Durbin model and other methods, data for the 28 cities in the Beijing–Tianjin–Hebei air pollution transmission channel over the period 2000–2017 are employed. Regarding data sources, haze pollution data are obtained from the Socioeconomic Data and Applications Center at Columbia University, which provides global annual mean PM_2.5_ concentration data derived from satellite observations. The dataset is available at https://www.earthdata.nasa.gov/centers/sedac-daac (accessed on 29 June 2026). Data on green patent grants are obtained from the China National Intellectual Property Administration and manually compiled by the authors. All other data are collected from the China Environmental Statistical Yearbook, China City Statistical Yearbook, China Urban Construction Statistical Yearbook, China Regional Economic Statistical Yearbook, and statistical yearbooks of individual provinces [31].

## 3. Statistical Analysis

### 3.1. Spatial Distribution Characteristics of Air Pollution-Intensive Industries

**Figure 1:**
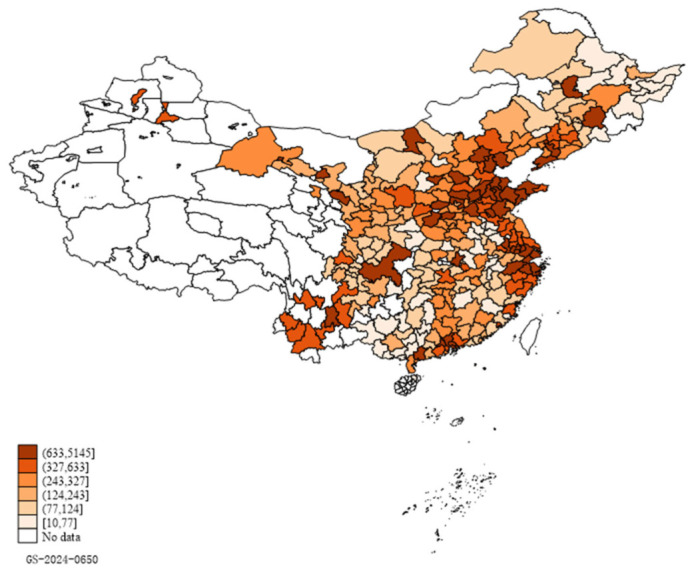
Quantile Map of Pollution-Intensive Industries (2006).

**Figure 2:**
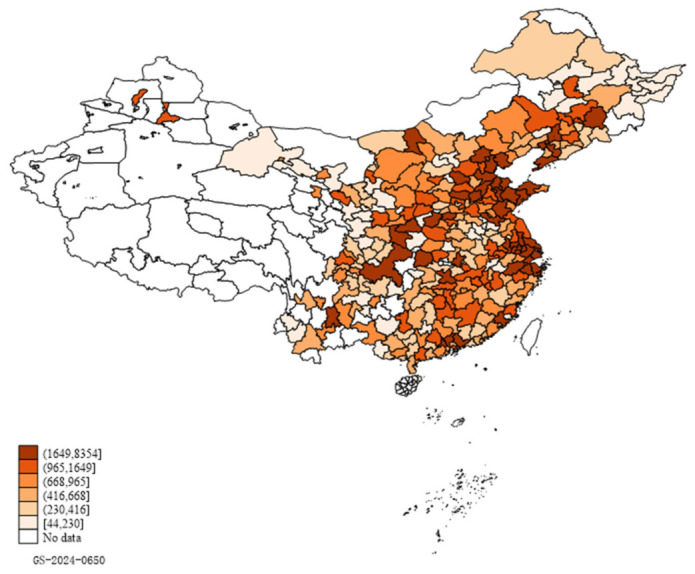
Quantile Map of Pollution-Intensive Industries (2012).

**Figure 3:**
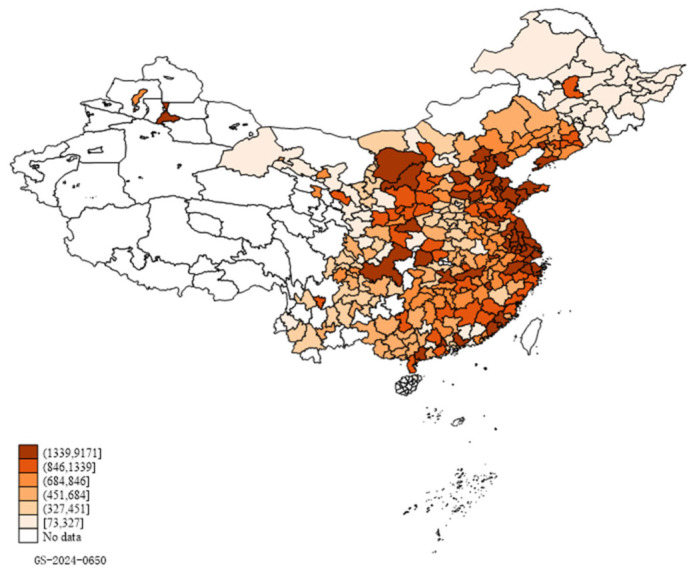
Quantile Map of Pollution-Intensive Industries (2018).

**Figure 4:**
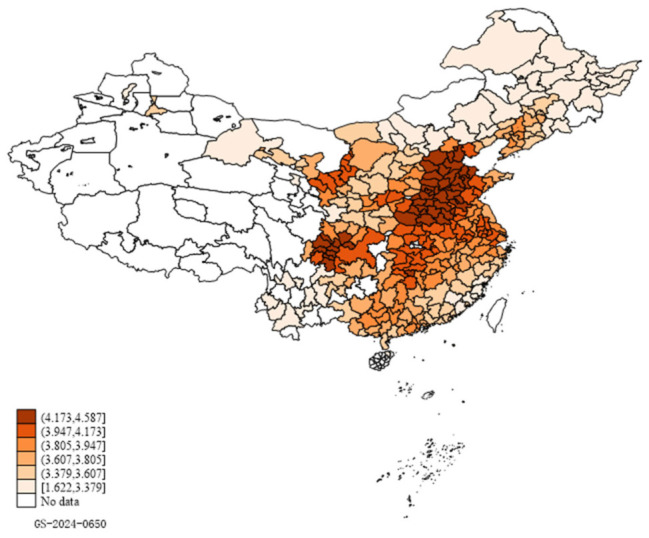
Quantile Map of Haze Pollution (2006).

**Figure 5:**
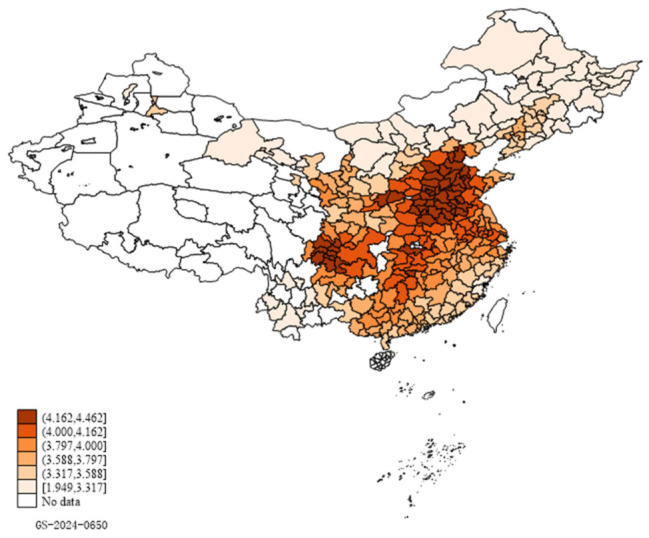
Quantile Map of Haze Pollution (2012).

**Figure 6:**
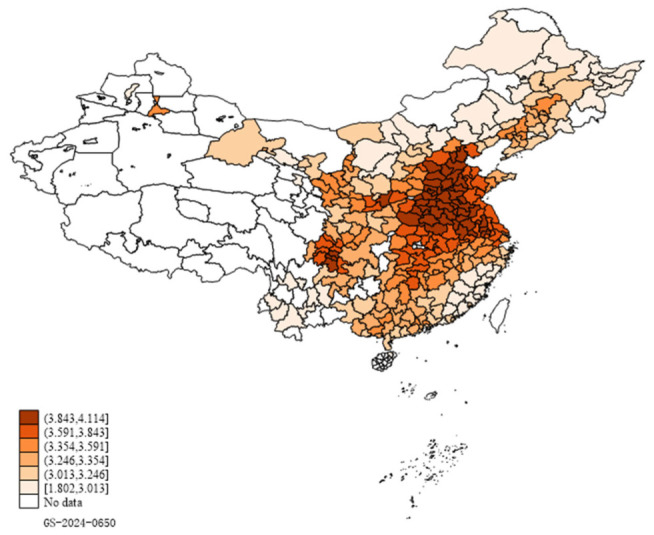
Quantile Map of Haze Pollution (2018).

The spatial distribution of air pollution-intensive industries is illustrated in Figure 1, Figure 2 and Figure 3. The spatial distribution of haze pollution is illustrated in Figure 4, Figure 5 and Figure 6.

Based on the changes in the distribution of pollution-intensive industries shown in Figure 1, Figure 2 and Figure 3 from 2006 to 2012 and 2018, and the changes in haze pollution shown in Figure 4, Figure 5 and Figure 6 over the same period, it can be observed that air pollution-intensive industries exhibit a pronounced pattern of spatial concentration. These industries are primarily clustered in the eastern and central regions of China, particularly in provinces with a strong industrial base such as Hebei, Shandong, Henan, and the Yangtze River Delta region. As a result, these areas are subject to relatively high environmental pressure. Notably, although Hebei, Shandong, and Henan host a relatively large concentration of air pollution-intensive industries, their atmospheric carrying capacity is relatively weak, leading to more severe haze pollution [32]. In contrast, while the Yangtze River Delta also exhibits a high concentration of such industries, its stronger atmospheric carrying capacity and more advanced environmental governance capacity mitigate the severity of haze pollution to some extent. From a temporal perspective, although the overall spatial pattern remains largely stable, notable changes can still be observed in certain regions. For instance, the output of air pollution-intensive industries in Hebei and Shandong shows a clear increasing trend over time, which is reflected in the deepening color intensity in the maps. In contrast, in some regions, color variations indicate industrial upgrading or relocation, reflecting a dynamic adjustment process in industrial structure. The spatial distribution of air pollution-intensive industries is closely aligned with China’s major economic zones. Concentrations are particularly evident in economically developed regions such as the Bohai Rim, the Yangtze River Delta, the Shandong Peninsula, and the Pearl River Delta. Meanwhile, government environmental regulations and industrial policies have exerted a significant influence on spatial redistribution, encouraging the relocation of highly polluting industries to regions with relatively lenient regulatory constraints or promoting technological upgrading within existing industrial bases. In addition, substantial regional disparities are observed. Due to relatively lagging economic development and weaker industrial foundations, western China hosts a significantly lower concentration of air pollution-intensive industries. Overall, the spatial distribution of air pollution-intensive industries is jointly shaped by economic development levels, industrial structure, and policy orientation. With continued economic growth and increasingly stringent environmental regulations, this spatial pattern is expected to undergo further transformation in the future.

### 3.2. Statistical Analysis of the Spatial Durbin Model

The statistical analysis results of the Spatial Durbin Model are as follows: The main regression results for the impact of output from pollution-intensive industries on haze pollution are presented in Table 3.

According to the SDM direct effect estimates, the output value of air pollution-intensive industries exerts a significantly positive impact on local haze pollution, with a coefficient of 0.028 * (note: *, **, and *** denote statistical significance at the 10%, 5%, and 1% levels, respectively). This suggests that an increase in the output level of such industries is associated with higher local haze pollution. This result can be attributed to the fact that increased output from pollution-intensive industries implies greater consumption of coal resources in production. During coal combustion, incomplete combustion and associated chemical reactions release large quantities of ultrafine particles. Furthermore, during end-of-pipe treatment processes such as desulfurization, denitrification, and dust removal, limitations inherent in the technologies themselves coupled with extensive management practices also lead to the generation of substantial amounts of UFPs.

Desulfurization in pollution-intensive industries: The limestone–gypsum wet flue gas desulfurization technology used in the desulfurization processes of pollution-intensive industries, combined with the mandatory lead-sealing of bypass flue gas dampers on desulfurization facilities in thermal power plants in 2010 and the nationwide abandonment of flue gas–gas heaters in thermal power plants in 2012, has resulted in the low-cost emission of large amounts of water vapor carrying enormous quantities of ultrafine particles [33,34,35].

Denitrification in pollution-intensive industries: In the denitrification process, the use of selective catalytic reduction (SCR) technology, together with the progressive and annual tightening of NO_x_ emission standards for denitrification, has led to secondary pollution mechanisms in the end-of-pipe treatment processes of pollution-intensive industries—such as ammonia slip and ammonium sulfate formation during wet desulfurization and denitrification, and the entrainment of sulfates and ultrafine particles from desulfurization wastewater evaporation with insufficient capture efficiency (typically below 50%). This has created a “treatment–emission paradox,” resulting in severe ammonia slip and a dramatic increase in secondary ultrafine particles [36,37]. More seriously, while generating ultrafine particles, these industrial production stages also release large amounts of water vapor. When water vapor combines with these ultrafine particles, haze forms more rapidly and disperses over a wider area. The resulting haze episodes are not only more intense but also more prolonged, causing greater harm to people’s daily lives and health [38].

Dust removal in pollution-intensive industries. To reduce the mass of particulate matter in the atmosphere, pollution-intensive industries have widely adopted electrostatic precipitation, a technology that is inefficient for fine particles, and this is a principal source of the abrupt increase in atmospheric ultrafine particles in China. The effectiveness of electrostatic precipitation is influenced by particle size: fine particles experience a weaker electric field force and are therefore difficult to collect. Electrostatic precipitation has been extensively deployed primarily to reduce particle mass; however, it targets mainly coarse particles and offers limited removal efficiency for ultrafine particles [39].

Typical case analysis: The cities with the most severe haze pollution in China from 2013 to 2016—Baoding, Xingtai, Tangshan, Shijiazhuang, Handan, and Jinan—were taken as examples for analysis. The sectors with the largest water vapor emissions in China are thermal power and iron and steel industries. The thermal power industry emits huge amounts of water vapor. For coal-fired power units with wet flue gas desulfurization that discharge saturated wet flue gas, each kilowatt-hour of electricity generated corresponds to a total water vapor emission of 2.15 kg. In 2017, the national thermal power generation was 4.66 trillion kWh. Preliminary estimates put water vapor emissions at 10.0 billion metric tons (100.0 × 10^8^ tons); considering that some units adopt air-cooled or once-through cooling methods, the estimated water vapor emissions are approximately 8.0 billion metric tons. Taking two 1000 MW coal-fired units (two 1000 MW units) as an example, assuming a daily average load factor of 75% in winter, the daily power generation is 36,000 MWh, corresponding to water vapor emissions of 77,400 tons. Under meteorological conditions of 0 °C ambient temperature, high humidity, and stagnant weather, and assuming an atmospheric boundary layer height of 500 m, the water vapor, if evenly dispersed, could raise the relative humidity of the air over an area of 310 km^2^ by 10% within one day. However, this water vapor is not evenly distributed: in a medium-sized city with a single large thermal power plant, if stagnant weather occurs, severe haze pollution will soon appear over most areas [2].

The iron and steel industry also discharges very large amounts of water vapor. Each ton of crude steel production corresponds to roughly 1.5 tons of water vapor emissions. Taking 2015 as an example, China’s crude steel output exceeded 800 million tons, corresponding to approximately 1.2 billion metric tons of water vapor emissions. Looking at the cities with the most severe haze pollution in China from 2013 to 2016—Baoding, Xingtai, Tangshan, Shijiazhuang, Handan, and Jinan—in 2015, Hebei Province’s crude steel output was 188 million tons; Tangshan City’s crude steel output was 82.697 million tons, discharging an average of 330,000 tons of water vapor per day; Handan City’s crude steel output was 43.5818 million tons, discharging an average of 179,100 tons of water vapor per day; and Jinan, Shijiazhuang, Baoding, and Xingtai each had a crude steel output of around 20 million tons, discharging an average of 82,100 tons of water vapor per day. If the water vapor emissions from crude steel production in these cities encounter stagnant weather, severe haze pollution can easily occur. Moreover, these cities all have multiple thermal power enterprises, and the water vapor and particulate matter emitted by these thermal power enterprises can also lead to severe haze pollution. During the heating season, thermal power enterprises discharge even greater amounts of water vapor and ultrafine particles, making the haze pollution even more severe [2].

The direct effect coefficient of carrying capacity for pollution-intensive industries in the SDM is −0.020 **, indicating a significant negative relationship with haze pollution. Further investigation reveals that this is because the atmospheric environmental system itself possesses a dynamic equilibrium and regulation mechanism for pollutants. In essence, the atmospheric environmental self-purification capacity is reflected in the carrying capacity for pollution-intensive industries, which defines the maximum dilution and dispersion limit for pollutants in a given region under specific meteorological conditions. When emissions exceed this threshold, the atmospheric chemical equilibrium is disrupted, accelerating the formation of secondary particles such as sulfate and nitrate, thereby increasing PM_2.5_ concentrations. If the atmospheric self-purification capacity is greater—for instance, under strong convection in summer or high wind speeds—pollutants are rapidly diluted and dispersed, and their concentrations can be stabilized within the allowable range of environmental capacity, making haze formation unlikely. At the core of the impact of atmospheric self-purification capacity on the environment is the natural purification process. Specifically, dry deposition uses gravity to cause particles to adhere to the surface, wet deposition removes aerosols through precipitation scavenging, and photochemical reactions convert gaseous pollutants into settleable particulate matter through oxidation. These mechanisms together constitute a dynamic pollutant removal system, and the operational efficiency of this system is directly related to the magnitude of the atmospheric self-purification capacity [28,40].

The coefficient of urbanization level is −0.136 ***, indicating a significant negative relationship with haze pollution. This result suggests that higher-quality urbanization is associated with lower pollution levels. This mechanism can be attributed to coordinated improvements in economic structure, technological progress, industrial upgrading, and environmental governance capacity. Specifically, improvements in energy structure and green technological innovation reduce emissions at the source; compact and polycentric urban development reduces transportation-related emissions; coordinated regional governance and smart environmental monitoring improve regulatory efficiency; and increased environmental awareness further reduces pollutant generation.

The indirect effect (spatial spillover) of pollution-intensive industrial output on haze pollution registers at 0.151 **, demonstrating statistical significance. Critically, the analysis reveals that increases in regional output within pollution-intensive industries significantly intensify haze pollution in neighboring regions through spatial spillover mechanisms [22]. This phenomenon can be explained by two main mechanisms. First, pollutants such as sulfur dioxide and ultrafine particles emitted during local heavy industry expansion, being lightweight and resistant to sedimentation, can easily drift with wind currents. These pollutants diffuse beyond regional borders via atmospheric circulation. Second, as industrial linkages extend supply chains upstream and downstream—spanning activities like raw material extraction and supporting logistics infrastructure—they indirectly elevate pollutant emissions in adjacent regions. This process establishes a chain of pollution transfer. These observations confirm a pronounced indirect effect linking pollution-intensive industries to haze pollution. The strong indirect effect identified underscores the significant spillover from local pollution-intensive industries which detrimentally impacts surrounding areas, thereby triggering regional haze pollution problems.

As shown in Table 2, the total effect of the output value of air pollution-intensive industries on haze pollution is significantly positive at 0.179 **. This result further confirms the strong positive association between industrial activity intensity and haze pollution at the regional level.

### 3.3. Multicollinearity Test and Spatial Correlation Test

#### 3.3.1. Multicollinearity Diagnostics

The multicollinearity test results (see Table 4) show that the variance inflation factor (VIF) of each explanatory variable is less than 10, with an average VIF of 3.27, indicating that the model as a whole does not suffer from serious multicollinearity problems.

#### 3.3.2. Spatial Weight Matrix Selection and LM Test Results

This paper adopts the Queen contiguity spatial weight matrix to construct spatial weights. Specifically, if two cities share a common boundary or a common vertex, they are considered to have a spatial adjacency relationship and the weight is assigned a value of 1; otherwise, it is 0. The weight matrix is then row-standardized. The choice of the Queen contiguity weight matrix is based on the following considerations. First, PM_2.5_ exhibits significant atmospheric dispersion characteristics. Its spatial transmission occurs not only between regions that share a common boundary, but also possibly through adjacent regions connected by common vertices. Therefore, compared with the Rook contiguity matrix, the Queen contiguity matrix can more comprehensively capture the spatial correlation between regions. Second, the spatial spillover effects of thermal power industry layout and pollution emissions are pronounced. Adjacent regions have strong mutual influences in terms of atmospheric flow, industrial linkages, and environmental governance. Adopting the Queen contiguity matrix is thus more consistent with the actual characteristics of pollution dispersion. Third, the Queen contiguity matrix is one of the most widely used contiguity weight matrices in spatial econometric research, with a solid theoretical foundation and extensive literature support. For these reasons, this paper uses this matrix as the baseline spatial weight matrix.

Moran’s I statistic indicates the presence of significant spatial dependence (*p* = 0.050). Furthermore, the robust LM error test result (See Table 5) is significant (*p* = 0.024), while the robust LM lag test result is near-significant (*p* = 0.052).

All empirical analyses in this study employ fixed-effects models. To determine the appropriate spatial econometric model, likelihood ratio (LR) tests were conducted to examine whether the spatial Durbin model (SDM) can be simplified to a spatial autoregressive model (SAR) or a spatial error model (SEM). The null hypothesis was rejected in both cases (LR = 16.88, *p* = 0.0182; LR = 16.43, *p* = 0.0215), indicating that the SDM cannot be reduced to either the SAR or the SEM. Therefore, the SDM is retained as the preferred model for subsequent analysis.

### 3.4. Robustness Checks

The robustness test results are reported in Table 6 and Table 7. Overall, the empirical findings indicate that the estimation results of the model are stable and reliable. To test robustness, the dependent variable (haze) is replaced by the Air Pollution Index (API) and SO_2_ to measure regional air pollution intensity. The spatial effect results based on this alternative proxy show that the estimated direct, indirect, and total effects are broadly consistent with those obtained in the baseline regressions. In particular, the total effect estimates indicate that, after substituting haze with the API, green technology innovation, administrative regional area, urbanization level, and registered urban unemployment rate are all significantly negatively associated with air pollution. These results can be interpreted as follows: First, improvements in green technology innovation are typically accompanied by more efficient pollution control technologies and wider application of clean energy. These advancements effectively reduce pollutant emissions, thereby lowering the API. Second, larger administrative regional areas are generally associated with greater ecological space and resource endowments. Such regions tend to exhibit stronger pollutant dilution and self-purification capacity. In addition, larger spatial scales allow for more flexible ecological planning and more rational industrial spatial allocation, which helps mitigate air pollution. Third, higher urbanization levels often reflect more advanced infrastructure and environmental management systems, including centralized heating and gas supply, improved wastewater and waste management systems, and more coordinated urban planning. These factors contribute to reduced emissions from dispersed pollution sources and therefore improve air quality. Finally, higher registered urban unemployment rates often indicate an economic slowdown, which may drive labor-intensive, high-emission industries to cut back or shut down. This reduces industrial emissions directly. Also, governments may increase environmental governance efforts to stabilize employment and improve urban competitiveness, further contributing to reductions in air pollution.

## 4. Research Conclusions and Policy Recommendations

### 4.1. Research Conclusions

In summary, pollution-intensive industries are important sources of UFPs and exert a significant promoting effect on haze pollution, with evident spatial spillover effects. The key conclusions are as follows:

First, the principal source of ultrafine particles (UFPs) in China’s haze pollution is desulfurization, denitrification, and dust removal processes within pollution-intensive industries (the direct effect of pollution-intensive industries on haze pollution is 0.028 *). Pollution-intensive industrial output exhibits a significantly positive correlation with haze pollution, indicating that an increase in local output of these industries significantly exacerbates local haze conditions. Desulfurization, denitrification, and electrostatic precipitation (ESP) processes in pollution-intensive industries systematically generate substantial UFP emissions. Extensive management practices in desulfurization, such as the nationwide decommissioning of gas–gas heaters (GGHs) in thermal power plants, have led to the massive, low-cost emission of water vapor carrying enormous quantities of UFPs. Escalating stringency in nitrogen oxide (NOx) emission standards has precipitated significant ammonia slip during wet desulfurization/denitrification processes, dramatically increasing secondary UFP formation. Crucially, the widespread adoption of ESP for reducing particulate matter mass—a technology notoriously inefficient at capturing fine particles—constitutes the main driver behind the abrupt surge of atmospheric UFPs in China.

Second, haze pollution in China predominantly affects regions densely concentrated with pollution-intensive industries yet possessing weak atmospheric self-purification capacity (the direct effect of atmospheric environmental carrying capacity on haze is −0.020 **). Atmospheric environmental carrying capacity exerts a significant negative influence on haze pollution. Pollution-intensive industries are predominantly concentrated in Hebei, Shandong, Henan, and the Yangtze River Delta region. Consequently, these regions bear relatively heavy environmental pollution pressure. Due to their relatively weak atmospheric self-purification capacity, Hebei, Shandong, and Henan suffer more severe haze pollution. In contrast, the Yangtze River Delta, benefiting from its stronger atmospheric self-purification capacity, experiences comparatively less severe haze pollution despite its high industrial concentration.

Third, another significant source of UFPs in China’s haze pollution originates from factors like airflow from neighboring regions (the indirect effect of pollution-intensive industries on haze pollution is 0.151 **). Pollution-intensive industries exhibit significant spatial spillover effects on haze pollution, meaning that an increase in local industrial output also significantly exacerbates haze pollution in adjacent regions.

### 4.2. Policy Recommendations

Based on the above, UFPs are primarily generated by pollution-intensive industries. Moreover, the impacts of these industries on haze pollution are significantly influenced by technological innovation and atmospheric carrying capacity. Therefore, a comprehensive governance framework covering the entire “emission–dispersion–deposition” process should be established from the perspectives of scientific regulation, regional coordination, and technological support, with an aim to reduce UFP concentrations in the atmosphere. The specific policy recommendations are as follows: First, promoting the gradient-based relocation of pollution-intensive industries according to differences in atmospheric self-purification capacity. Industrial transfer pathways should be designed in accordance with regional self-purification capacity levels, supported by indicators such as pollution-intensive industry gradient coefficients, dynamic industrial agglomeration indices, and feasibility assessments of industrial relocation. This would enable a scientifically guided redistribution of high-emission industries. Second, systematically improving flue gas desulfurization processes in pollution-intensive industries. This includes restoring the function of gas-to-gas heat exchangers where environmentally necessary to increase exhaust temperature, enhancing wet desulfurization technologies, accelerating the development and substitution of dry desulfurization technologies, and thereby improving overall emission control efficiency. Third, strengthening technological innovation in denitrification processes and strictly regulating ammonia emissions. This can be achieved by establishing more scientific nitrogen oxide emission standards, developing reliable ammonia slip monitoring technologies for complex flue gas environments, optimizing monitoring site selection, and exploring alternative oxidants beyond ammonia to reduce secondary pollution formation. Fourth, enhancing research and development of core UFP removal technologies. Integrated approaches combining electrostatic precipitation with agglomeration techniques and hybrid systems combining bag filtration with electrostatic methods should be promoted to improve overall particulate removal efficiency. Fifth, strengthen regional collaborative governance through systematic enhancement of the Joint Prevention and Control (JPC) system. This requires: establishing a comprehensive inter-regional cooperation framework, implementing targeted training programs for administrative personnel, and scientifically elevating environmental management competencies. These coordinated measures are essential to effectively mitigate cross-jurisdictional pollution spillover effects.

## Figures and Tables

**Table 1:** Variable Definitions and Measurements.

Variable Type	Variable	Naming
Dependent Variable	Haze	PM_2.5_
	Air pollution index	pollution
	SO_2_	lnSO_2_
Key Explanatory Variable	Output value of air pollution-intensive industries	output1
	Sales value of air pollution-intensive industries	output2
Threshold Variable	Carrying Capacity Index for Pollution-Intensive Industries	carrying
Control Variable	Green technological innovation	gti
	Administrative area size	K _1_
	Urbanization level	K _2_
	Registered urban unemployment rate	K _3_
	Tax policy	K _4_

Variables are defined as follows: Haze: PM_2.5_, as a key component of haze, plays a central role in its impacts on human production and daily life. Zhang et al. (2023) conducted an in-depth study on the distribution characteristics of PM_2.5_ under haze conditions [19]. In the assessment of haze pollution, PM_2.5_ concentration is a commonly used indicator, as its magnitude can directly and accurately reflect the intensity of haze pollution.

**Table 2:** Evaluation Indicator System for Carrying Capacity of Pollution-Intensive Industries.

Dimension	Indicator	Dimension	Indicator
Industrial Attractiveness	Average wage of employees (yuan)	Industrial Selectivity	Cumulative precipitation
	Number of foreign-invested enterprises (units)		Average temperature
	Total fixed asset investment (10,000 yuan)		Average 2 min wind speed
	Number of industrial enterprises above designated size (units)		Annual maximum wind speed
	Total fixed assets of industrial enterprises above designated size (10,000 yuan)		Maximum daily precipitation
	Amount of foreign capital actually utilized in the current year (10,000 US dollars)		Number of days with daily precipitation ≥ 0.1 mm
	Year-end loan-to-deposit ratio of financial institutions		Average atmospheric pressure
Industrial Development Capacity	Proportion of value added of secondary industry in GDP (%)		Park green space area (hectares, municipal district)
	Proportion of value added of tertiary industry in GDP (%)		Green coverage rate of built-up areas (%)
	Education expenditure (10,000 yuan)		Population density
	Number of students enrolled in regular higher education institutions (persons)		Built-up area
Industrial Support Capacity	Per capita gross regional product (yuan)		
	Local government general budgetary revenue (10,000 yuan)		
	Industrial wastewater discharge (10,000 tons)		
	Total postal business volume(10,000 yuan)		
	Natural population growth rate (‰)		
	Total retail sales of consumer goods (10,000 yuan)		

**Table 3:** Direct Effects of Industrial Output on Haze Pollution.

	(1) SAR		(2) SDM	(1) SAR	(2) SDM	(1) SAR	(2) SDM
	lnpm25		lnpm25	lnpm25	lnpm25	lnpm25	lnpm25
LR_Direct	Direct Effects			Indirect Effects		Total Effects	
lnoutput1	0.023		0.028 *	0.040	0.151 **	0.062	0.179 **
	(0.016)		(0.016)	(0.028)	(0.063)	(0.044)	(0.071)
carrying	−0.021 **		−0.020 **	−0.036 **	−0.075	−0.057 **	−0.095 *
	(0.010)		(0.010)	(0.018)	(0.052)	(0.028)	(0.055)
lngti	0.001		−0.005	0.002	−0.072	0.002	−0.077
	(0.018)		(0.017)	(0.032)	(0.073)	(0.050)	(0.078)
lnk1	−0.319		−0.408	−0.581	−1.836	−0.900	−2.244
	(0.282)		(0.326)	(0.538)	(1.619)	(0.815)	(1.862)
lnk2	−0.164 ***		−0.136 ***	−0.291 ***	−0.183	−0.455 ***	−0.319
	(0.046)		(0.049)	(0.088)	(0.186)	(0.131)	(0.197)
lnk3	−0.041		−0.028	−0.072	−0.080	−0.113	−0.108
	(0.027)		(0.027)	(0.049)	(0.126)	(0.075)	(0.141)
lnk4	0.009		−0.036	0.016	0.131	0.025	0.095
	(0.030)		(0.032)	(0.054)	(0.136)	(0.084)	(0.145)
N	364		364	364	364	364	364
*R*^2^_o	0.051		0.061	0.051	0.061	0.051	0.061
*R*^2^_w	0.386		0.500	0.386	0.500	0.386	0.500
*R*^2^_b	0.084		0.094	0.084	0.094	0.084	0.094
ll	328.623		337.061	328.623	337.061	328.623	337.061

Standard errors in parentheses. * *p* < 0.1, ** *p* < 0.05, *** *p* < 0.01.

**Table 4:** Multicollinearity Test.

Variable	VIF	1/VIF
lnoutput1	2.3	0.434
carrying	2.9	0.345
lngti	5.62	0.178
lnk1	1.22	0.819
lnk2	1.75	0.572
lnk3	1.21	0.830
lnk4	7.9	0.127
Mean VIF	3.27	

**Table 5:** LM Test for Spatial Correlation.

Test	Statistic	df	*p*-Value
Spatial error:			
Moran’s I	1.957	1.000	0.050
Lagrange multiplier	3.066	1.000	0.080
Robust Lagrange multiplier	5.076	1.000	0.024
Spatial lag:			
Lagrange multiplier	1.761	1.000	0.185
Robust Lagrange multiplier	3.771	1.000	0.052

**Table 6:** Robustness regression results with the Air Pollution Index as the dependent variable.

	(1) SAR	(2) SDM	(1) SAR	(2) SDM	(1) SAR	(2) SDM
	lnpm25	lnpm25	lnpm25	lnpm25	lnpm25	lnpm25
LR_Direct	Direct Effects		Indirect Effects		Total Effects	
lnoutput1	0.146 *	0.117 *	0.090 *	0.707 ***	0.237 *	0.824 ***
	(0.080)	(0.067)	(0.050)	(0.183)	(0.128)	(0.209)
carrying	−0.136 ***	−0.100 **	−0.085 **	0.205	−0.221 ***	0.105
	(0.049)	(0.042)	(0.033)	(0.151)	(0.080)	(0.156)
lngti	−0.226 **	−0.233 ***	−0.140 **	−0.566 ***	−0.366 ***	−0.799 ***
	(0.088)	(0.075)	(0.057)	(0.217)	(0.141)	(0.231)
lnk1	0.250	−1.925	0.141	−11.154 **	0.391	−13.079 **
	(1.377)	(1.311)	(0.884)	(4.618)	(2.253)	(5.480)
lnk2	−0.028	0.568 ***	−0.016	−2.142 ***	−0.044	−1.575 ***
	(0.225)	(0.210)	(0.145)	(0.546)	(0.369)	(0.565)
lnk3	−0.334 **	−0.109	−0.209 **	−0.764 **	−0.543 **	−0.873 **
	(0.130)	(0.112)	(0.089)	(0.357)	(0.214)	(0.404)
lnk4	−0.365 **	−1.237 ***	−0.228 **	1.150 ***	−0.593 **	−0.087
	(0.145)	(0.137)	(0.097)	(0.406)	(0.236)	(0.423)
N	364	364	364	364	364	364

Standard errors in parentheses. * *p* < 0.1, ** *p* < 0.05, *** *p* < 0.01.

**Table 7:** Robustness regression results with SO_2_ as the dependent variable.

	(1) SAR	(2) SDM	(1) SAR	(2) SDM	(1) SAR	(2) SDM
	lnpm25	lnpm25	lnpm25	lnpm25	lnpm25	lnpm25
LR_Direct	Direct Effects		Indirect Effects		Total Effects	
lnoutput1	0.163 **	0.170 **	0.155 **	0.640 ***	0.318 **	0.810 ***
	(0.081)	(0.074)	(0.078)	(0.199)	(0.157)	(0.229)
carrying	−0.196 ***	−0.175 ***	−0.189 ***	0.087	−0.386 ***	−0.088
	(0.050)	(0.046)	(0.055)	(0.168)	(0.100)	(0.173)
lngti	−0.174 **	−0.175 **	−0.167 *	−0.703 ***	−0.340 *	−0.878 ***
	(0.089)	(0.082)	(0.089)	(0.238)	(0.174)	(0.253)
lnk1	−0.086	−2.332	−0.106	−11.541 **	−0.192	−13.873 **
	(1.404)	(1.445)	(1.398)	(5.082)	(2.792)	(6.027)
lnk2	−0.251	0.156	−0.238	−1.912 ***	−0.489	−1.756 ***
	(0.232)	(0.232)	(0.227)	(0.601)	(0.456)	(0.622)
lnk3	−0.361 ***	−0.208 *	−0.349 **	−0.744 *	−0.710 ***	−0.952 **
	(0.132)	(0.123)	(0.140)	(0.393)	(0.266)	(0.445)
lnk4	0.016	−0.643 ***	0.017	1.362 ***	0.033	0.719
	(0.148)	(0.151)	(0.147)	(0.446)	(0.293)	(0.465)
*N*	364	364	364	364	364	364
*R*^2^_o	0.047	0.003	0.047	0.003	0.047	0.003
*R*^2^_w	0.446	0.614	0.446	0.614	0.446	0.614
*R*^2^_b	0.010	0.004	0.010	0.004	0.010	0.004
ll	−268.872	−230.837	−268.872	−230.837	−268.872	−230.837

Standard errors in parentheses. * *p* < 0.1, ** *p* < 0.05, *** *p* < 0.01.

## Data Availability

The original contributions presented in this study are included in the article. Further inquiries can be directed to the corresponding author.
